# Premorbid use of selective beta-blockers improves sepsis incidence and course: Human cohort and animal model studies

**DOI:** 10.3389/fmed.2023.1105894

**Published:** 2023-04-18

**Authors:** Shiao-Ya Hong, Chih-Cheng Lai, Nai-Chi Teng, Chao-Hsien Chen, Chun-Chun Hsu, Nai-Ju Chan, Cheng-Yi Wang, Ya-Hui Wang, You Shuei Lin, Likwang Chen

**Affiliations:** ^1^Department of Biotechnology and Laboratory Science in Medicine, National Yang Ming Chiao Tung University, Taipei City, Taiwan; ^2^Medical Research Center, Cardinal Tien Hospital, New Taipei City, Taiwan; ^3^Division of Hospital Medicine, Department of Internal Medicine, Chi Mei Medical Center, Tainan City, Taiwan; ^4^Institute of Population Health Sciences, National Health Research Institutes, Miaoli County, Taiwan; ^5^Division of Pulmonary Medicine, Department of Internal Medicine, MacKay Memorial Hospital, Taipei City, Taiwan; ^6^Department of Medicine, MacKay Medical College, New Taipei City, Taiwan; ^7^School of Respiratory Therapy, College of Medicine, Taipei Medical University, Taipei City, Taiwan; ^8^Graduate Institute of Medical Sciences, College of Medicine, Taipei Medical University, Taipei City, Taiwan; ^9^Department of Internal Medicine, Cardinal Tien Hospital and School of Medicine, College of Medicine, Fu Jen Catholic University, New Taipei City, Taiwan; ^10^Department of Physiology, School of Medicine, College of Medicine, Taipei Medical University, Taipei City, Taiwan

**Keywords:** sepsis, beta-blockers, hypertension, atenolol, PD-L1

## Abstract

**Introduction:**

Beta-blockers are widely prescribed to manage hypertension and cardiovascular diseases and have been suggested as an attractive therapy to improve the prognosis of sepsis. Herein, we investigated the potential benefits of premorbid selective beta-blocker use in sepsis with a real-world database and explored the underlying mechanism by *in vivo* and *in vitro* experiments.

**Methods:**

A total of 64,070 sepsis patients and 64,070 matched controls who were prescribed at least one anti-hypertensive drug for more than 300 days within 1 year were selected for the nested case–control study. Female C57BL/6 J mice and THP-1 cells stimulated with lipopolysaccharide (LPS) were used for studying systemic responses during sepsis to validate our clinical findings.

**Results:**

The risk of sepsis was lower in current selective beta-blocker users than in non-users (adjusted OR (aOR), 0.842; 95% CI, 0.755–0.939), and in recent users than in non-users (aOR, 0.773; 95% CI, 0.737–0.810). A mean daily dose of ≥0.5 DDD was associated with a lower risk of sepsis (aOR, 0.7; 95% CI, 0.676–0.725). Metoprolol, atenolol, and bisoprolol users had lower risk of sepsis than non-users. In a LPS-induced sepsis mouse model, mice pre-fed with atenolol had significantly reduced mortality. While atenolol had some mild effects on LPS-induced release of inflammatory cytokines in septic mice, it significantly reduced serum soluble PD-L1 levels. Notably, atenolol treatment reversed the negative correlation of sPD-L1 with inflammatory cytokines in septic mice. Moreover, atenolol markedly downregulated the PD-L1 expression on LPS-stimulated THP-1 monocytes/macrophages *via* targeting ROS-induced NF-κB and STAT3 activation.

**Conclusion:**

Atenolol pretreatment can reduce sepsis mortality in mice, and *in vivo* and *in vitro* studies of PD-L1 expression suggest a role for atenolol in the modulation of immune homeostasis. These findings may contribute to the reduced incidence of sepsis in hypertensive patients with premorbid treatment with selective beta-blockers, especially atenolol.

## 1. Introduction

Sepsis is an overwhelming and life-threatening complication caused by infection (1). Despite recent significant advances in intensive care medicine, sepsis is still one of the leading causes of death in the intensive care unit, affecting approximately 49 million people and causing 11 million deaths globally every year (2). To manage this global burden and improve the outcomes for patients with sepsis, the Surviving Sepsis Campaign initiative was developed in 2004, and issues periodically updated professional guidelines (3–5). However, morbidity and mortality attributed to sepsis remains high (6, 7). Therefore, investigation into other potential or novel treatments for sepsis is urgently needed.

A number of medications are used for treating sepsis and septic shock. Rapid administration of broad-spectrum antibiotics, intravenous fluids, vasopressors, and active restoration of tissue oxygen delivery reduce mortality in patients with sepsis (8). Other medications for sepsis include low doses of corticosteroids, insulin to help maintain stable blood sugar levels, drugs that modify immune system responses, and painkillers or sedatives. However, patients with sepsis often suffer fever, shock, and respiratory failure due to an uncontrolled pro-inflammatory response known as systemic inflammatory response syndrome, resulting in approximately a quarter of sepsis patients to die (9). Among the patients who survive cytokine storm, one-third recover their immunity, with a mortality rate of 10%, while the other two-thirds develop immunoparalysis, accounting for 65% of total mortality (10). The high mortality and morbidity in sepsis patients is now thought to result from the sepsis-induced immunoparalysis, which hinders the clearance of the primary infection and renders patients more susceptible to secondary infection with opportunistic pathogens.

Beta-blockers are a class of anti-hypertensive medications that work by temporarily stopping or reducing the action of the beta-adrenergic receptors (11). They are prevalently used to manage a variety of cardiovascular disorders, including congestive heart failure, arrhythmia, and coronary artery disease. In addition to the well-known beneficial effects of beta-blockers on hemodynamics, beta-blockers can be involved in the modulation of cellular immune functions through affecting the adrenergic system (11). In a meta-analysis by Tank et al., nine studies comprising 56,414 patients with sepsis including 6,576 patients with premorbid exposure to beta-blockers were analyzed. The study showed that beta-blocker exposure prior to sepsis is associated with reduced mortality (12, 13). Chronic prescription of beta-blockers has also been reported to effectively improve cardiac rhythm and acid–base parameters, and reduce the mortality rate in patients with sepsis (14). The immunomodulatory properties of beta-blockers have been demonstrated (15–18) and these proposed immunologic effects may make beta-blockers drugs that are potentially useful for sepsis (19, 20). Recent evidence also suggests that premorbid beta-blockers may be an attractive therapy to improve the prognosis of sepsis (21–24). However, clinical studies have not shown consistent results regarding the beneficial effects of individual beta-blockers for sepsis (25–29).

Considering the lack of large-scale studies addressing this issue, further research to prove the beneficial effects of beta-blockers for sepsis is urgently needed. Under this premise, this study aimed to investigate the effect of beta-blockers including selective and non-selective beta-blockers on the development of sepsis in a nationwide population, and furthermore attempt to validate the real-world database findings through conducting *in vitro* and *in vivo* experiments. We further tried to understand the impact of premorbid beta-blocker use on the molecular changes during sepsis onset.

## 2. Materials and methods

### 2.1. Study population and ethical approval

Using the nationwide National Health Insurance Research Database (NHIRD) of Taiwan, we conducted a population-based nested case–control study. The study population consisted of 2.2 million subjects with heart or lung disease who held Taiwan National Health Insurance. Complete outpatient and inpatient electronic claim records, individual diagnoses, surgical procedures, and prescribed medications are available in the NHIRD database. The study cohort consisted of hypertension patients taking at least one anti-hypertensive drug for more than 300 days within 1 year. All patients who were longitudinally followed from the NHIRD between January 2000 and December 2010 and who were aged 18 years or older on 1 January 2000 were eligible for inclusion in this study. The study was approved on 17 December 2012 by the Institutional Review Board of the National Health Research Institutes (Taiwan) with a waiver of informed consent (protocol no. EC1011008-E). All procedures were followed in accordance with the ethical standards of the Institutional Review Board of the National Health Research Institutes and with the Helsinki Declaration of 1975.

### 2.2. Study design

We identified newly diagnosed sepsis by ICD-9-CM codes as evidence of bacterial or fungal infection and acute organ dysfunction (30–33). This definition of sepsis cases has been used in previous studies and was validated in a linked survey database. The index date referred to the first date of sepsis diagnosis. The one-year period preceding the index date was used for the assessment of beta-blocker exposure status. For each case, one control was randomly selected using the incidence density sampling method and matched by index date, 5-year age group, and sex. Nonselective beta-blockers (ATC code C07AA) and selective beta-blockers (ATC code C07AB) dispensed before the index date were identified from the NHIRD, and drug exposure was defined by having a drug prescription for at least 7 days. Exposure was defined using four different time frames. Current user status referred to patients with a selective or nonselective beta-blocker prescription that was filled within 90 days of the index date. Recent user status referred to patients with a selective or nonselective beta-blocker prescription filled between 90 and 365 days prior to the index date.

### 2.3. Animal study

Seven-week-old female C57BL/6 J mice (20–25 g) were randomized into two groups: mice were pretreated with normal water or 0.375 mg/mL atenolol in drinking water (equivalent to ~75 mg/kg/day based on 5 mL daily water intake) for 14 days (n = 20 in each group). Mice were injected intraperitoneally with a single dose of lipopolysaccharide (LPS, 15 mg/kg; *E. coli* 055: B5 purchased from Sigma-Aldrich) on day 15 to induce sepsis as previously described (34). The survival rate of the LPS-induced septic mice was compared between the control group and the study group administrated with atenolol. At 12 h post-LPS injection, their inflammatory cytokines and immunomodulatory checkpoint molecules in serum were measured by the ProcartaPlex multiplex assay (Level Biotechnology, Taiwan) and the PD-L1 expression in F4/80 positive cells was detected using flow cytometry. All operations were executed in compliance with the applicable regulations. This study was approved by the Institutional Animal Care and Use Committee of Cardinal Tien Hospital, New Taipei, Taiwan (protocol no. 107A-006).

### 2.4. Cell culture and treatments

Human monocyte THP-1 cells (RRID: CVCL_0006) were obtained from ATCC and cultured in RPMI 1640 medium (Gibco, Invitrogen), containing 10% heat-inactivated fetal bovine serum (FBS), 2 mM L-glutamine, 20 IU/mL penicillin, 20 μg/mL streptomycin, and 0.05 mM 2-mercaptoethanol. Human monocyte-derived macrophages were differentiated from THP-1 cells by culturing for 7 days in completed RPMI 1640 medium containing 20 ng/mL 12-O-tetradecanoylphorbol-l3-acetate (PMA). Cells were pretreated with or without atenolol for 7 days and then stimulated with or without 100 ng/mL LPS, as indicated.

### 2.5. ROS detection and phagocytosis assay

For ROS detection, cells (5 × 10^5^) were incubated with 5 μM H2DCFDA dye (Invitrogen, NY, USA) in 6-well plates at 37°C in a 5% CO2 incubator for 30 min. Following removal of the H2DCFDA dye, cells were collected by centrifugation at 300 g, resuspended in PBS, and then detected by flow cytometry. The positive rate of reactive oxygen species (ROS) was analyzed and computed using the Cytobank software (Cytobank, Beckman Coulter). For phagocytosis assay, cells were co-cultured with 100 μg pHrodo-conjugated Escherichia coli (Invitrogen, NY, USA) at 37°C for 2 h. Cells were washed with PBS, harvested by centrifugation, and detected by flow cytometry. The percentages of pHrodo-positive cells were analyzed using the Kaluza software (Beckman Coulter).

### 2.6. Western blot analysis

Cells were lysed with a RIPA buffer containing 150 mM NaCl, 50 mM Tris–HCL, 0.1% SDS, 1% Triton X-100, 0.5% Na deoxycholate, 2 mM EDTA, and 1× protease/phosphatase inhibitor cocktail (Roche Diagnostic). Whole cell lysates were collected by centrifugation at 13,000 rpm, heated in sample buffer at 95°C for 5 min, separated by SDS electrophoresis, and then transferred to nitrocellulose membranes by semi-dry electroblotting. The membranes were blocked with 5% non-fat milk in Tris-buffered saline containing 0.1% Tween 20 (TBST), and incubated overnight at 4°C with the corresponding primary antibodies. The membranes were then washed with TBST, incubated with horseradish peroxidase-conjugated secondary antibodies, washed again, and developed with enhanced chemiluminescence. The images were acquired by an Imaging System. All antibodies used in this study (PD-L1 Cat# 13684, RRID: AB_2687655; phospho-NF-κB p65 Cat# 3033, RRID: AB_331284; NF-κB p65 Cat# 8242, RRID: AB_10859369; phospho-Stat3 Cat# 9145, RRID: AB_2491009; STAT3 Cat# 9139, RRID: AB_331757; β-actin Cat# 3700, RRID: AB_2242334) were obtained from Cell Signaling Technology (MA, United States).

### 2.7. Reverse transcription quantitative polymerase chain reaction

Total RNA was extracted from cells with the Genezol reagent (Genaid, Taiwan) and was submitted to reverse transcription using the TAKARA cDNA Reverse Transcription kit (Takara, Japan). Quantitative PCR was performed with the fluorescent dye SYBR Green methodology and a Rotor-Gene Q real-time PCR system. Primers for gene expression analysis were purchased from IDT (Singapore). These mean Cq values were used to normalize the steady-state target mRNA concentrations to those of the beta-actin by the 2(−ΔΔCq) method.

### 2.8. Statistical analysis

Data were expressed as mean and standard deviation or numbers with percentages. We compared the baseline characteristics between case and control groups by Student’s t test or chi-squared test according to the type of variables. Conditional logistic regression analysis was used to estimate type, dosage and duration of beta-blockers with regard to the risk of sepsis. The crude and adjusted odds ratios (ORs) and 95% confidence intervals (CIs) were calculated with conditional logistic regression. ORs were adjusted for Charlson Comorbidity Index (CCI), age, and the use of other antihypertensive drugs. On the basis of the duration of beta-blocker treatment, we divided the patients into current users (≤ 90 days) and past users (91–365 days) and took nonusers as the reference. The mean daily dosage of beta-blocker treatment was calculated based on defined daily dose (DDD) divided by the cumulative period, and divided into two categories as less than 0.5 DDD/d and more than 0.5 DDD/d. A two-sided *p* value of less than 0.05 was considered statistically significant. SAS version 9.4 (SAS Institute, Cary, NC, United States) and GraphPad Prism Software (San Diego, CA, USA) were used for the analyses.

## 3. Results

### 3.1. The associations between the type and dosage of beta-blockers and the risk of sepsis

Between 2000 and 2010, a total of 64,070 cases diagnosed with sepsis were identified from the NHIRD and matched to another 64,070 control subjects. The case group had more underlying comorbidities, including acute myocardial infarction, congestive heart failure, peripheral vascular disease, stroke, dementia, rheumatologic disease, peptic ulcer disease, renal disease, liver disease, malignancy and diabetes mellitus; and higher CCI scores than the control group (Table 1). The associations between the use of selective and nonselective beta-blockers and the risk of sepsis are summarized in Table 2. For the selective beta-blocker users, current users were associated with a lower risk of sepsis than the non-users (adjusted OR (aOR), 0.842; 95% CI, 0.755–0.939), and this trend was also observed with the recent users (aOR, 0.773; 95% CI, 0.737–0.810). In addition, the mean daily dosage ≥0.5 DDD was associated with a lower risk of sepsis (aOR, 0.7; 95% CI, 0.676–0.725), but the risk of sepsis was not changed by beta-blockers at the mean daily dosage <0.5 DDD (aOR, 1.009; 95% CI, 0.97–1.051). In contrast, for the nonselective beta-blocker users, current users were associated with a higher risk of sepsis than non-users (aOR, 1.246; 95% CI, 1.141–1.36), but recent users were associated with a lower risk of sepsis than non-users (aOR, 0.955; 95% CI, 0.914–0.997). A higher risk of sepsis among nonselective beta-blocker users than non-users was observed at different dosages. The risk of sepsis among patients receiving different selective and nonselective beta-blockers is shown in Table 3. Among the six selective beta-blockers, metoprolol, atenolol and bisoprolol were found to be associated with lower risks of sepsis than non-users (metoprolol, aOR, 0.767; 95% CI, 0.607–0.967; atenolol, aOR, 0.915; 95% CI, 0.840–0.997; bisoprolol, aOR, 0.922; 95% CI, 0.852–0.998). For the other three selective beta-blockers, betaxolol, acebutolol and esmolol, the risks of sepsis were similar to control groups. However, the risk of sepsis did not vary among individual nonselective beta-blockers.

**Table 1:** Baseline characteristics of sepsis cases and their matched controls selected from a cohort of patients with hypertension.

Variables	Cases (*n* = 64,070)	Controls (*n* = 64,070)
Age, year (SD)	71.89 (11.25)	71.91 (11.27)
Period, years (SD)	3.81 (2.49)	3.81 (2.49)
Male Gender, *n* (%)	34,683 (54.13)	34,683 (54.13)
Charlson Score (SD)	2.39 (1.97)	1.42 (1.63)
*Baseline Comorbidities, n (%)*		
Myocardial infarction	2026 (3.16)	1,487 (2.32)
Congestive heart failure	11,079 (17.29)	6,037 (9.42)
Peripheral vascular disease	1,559 (2.43)	1,094 (1.71)
Cerebrovascular disease	13,156 (20.53)	8,467 (13.22)
Dementia	6,533 (10.20)	3,604 (5.63)
Rheumatologic disease	928 (1.45)	743 (1.16)
Peptic ulcer disease	13,571 (21.18)	10,116 (15.79)
Hemiplegia or paraplegia	1,278 (1.99)	633 (0.99)
Renal disease	10,209 (15.93)	4,828 (7.54)
Moderate or severe liver disease	4,363 (6.81)	3,069 (4.79)
Tumor	6,745 (10.53)	4,392 (6.86)
Diabetes	23,057 (35.99)	16,455 (25.68)

**Table 2:** The association between the use of beta-blockers and the risk of sepsis.

Type of beta-blockers	Controls (*n* = 64,070)*n*(%)	Cases (*n* = 64,070)*n*(%)	Crude OR (95% CI)	Adjusted OR^#^ (95% CI)
*Selective*				
Non-user	46,245 (72.18%)	46,918 (73.23%)	Reference	Reference
*All user*				
Current user (≤ 90 days)	12,863 (20.08%)	12,017 (18.76%)	0.907 (0.831–0.990)	0.842 (0.755–0.939)
Recent user (91–365 days)	4,962 (7.74%)	5,135 (8.01%)	1.019 (0.978–1.062)	0.773 (0.737–0.810)
*Mean daily dosage*				
< 0.5 DDD/d	6,358 (9.92%)	7,986 (12.46%)	1.239 (1.196–1.284)	1.009 (0.970–1.051)
≥ 0.5 DDD/d	11,467 (17.90%)	9,166 (14.31%)	0.781 (0.757–0.805)	0.700 (0.676–0.725)
*Nonselective*				
Non-user	46,893 (73.19%)	38,584 (60.22%)	Reference	Reference
*All user*				
Current user (≤ 90 days)	11,185 (17.46%)	19,067 (29.76%)	1.580 (1.466–1.704)	1.246 (1.141–1.360)
Recent user (91–365 days)	5,992 (9.35%)	6,419 (10.02%)	1.302 (1.253–1.353)	0.955 (0.914–0.997)
*Mean daily dosage*				
< 0.5 DDD/d	6,482 (10.12%)	12,798 (19.98%)	2.433 (2.352–2.517)	1.861 (1.793–1.932)
≥ 0.5 DDD/d	10,695 (16.69%)	12,688 (19.80%)	1.443 (1.400–1.486)	1.163 (1.125–1.202)

# Adjusted for Charlson Score, age, and the use of other antihypertension drugs.

**Table 3:** The association between the use of selective and nonselective beta-blockers and the risk of sepsis.

Type	Control (*n* = 64,070)*n* (%)	Case (*n* = 64,070)*n* (%)	Crude OR (95% CI)	Adjusted OR^#^ (95% CI)
*Selective*				
Atenolol	5,096 (7.95%)	3,986 (6.22%)	0.825 (0.758–0.897)	0.915 (0.840–0.997)
Bisoprolol	4,988 (7.79%)	4,756 (7.42%)	0.885 (0.818–0.956)	0.922 (0.852–0.998)
Metoprolol	737 (1.15%)	550 (0.86%)	0.719 (0.572–0.905)	0.767 (0.607–0.967)
Acebutolol	428 (0.67%)	259 (0.40%)	0.937 (0.678–1.294)	1.010 (0.728–1.399)
Betaxolol	309 (0.48%)	248 (0.39%)	0.675 (0.468–0.972)	0.753 (0.521–1.089)
Esmolol	38 (0.06%)	65 (0.10%)	0.639 (0.287–1.421)	0.613 (0.273–1.377)
*Nonselective*				
Propranolol	3,752 (5.86%)	4,069 (6.35%)	1.028 (0.916–1.154)	1.020 (0.908–1.146)
Carteolol	104 (0.16%)	73 (0.11%)	0.729 (0.347–1.530)	0.721 (0.341–1.522)
Nadolol	79 (0.12%)	85 (0.13%)	1.077 (0.506–2.291)	1.114 (0.521–2.381)
Alprenolol	37 (0.06%)	43 (0.07%)	1.000 (0.323–3.101)	1.099 (0.352–3.429)
Sotalol	13 (0.02%)	13 (0.02%)	1.000 (0.141–7.099)	0.832 (0.117–5.933)

# Adjusted for Charlson Score, age, and the use of other antihypertension drugs.

### 3.2. Atenolol administration improved the survival of septic mice by modulating the immune homeostasis

**Figure 1:**
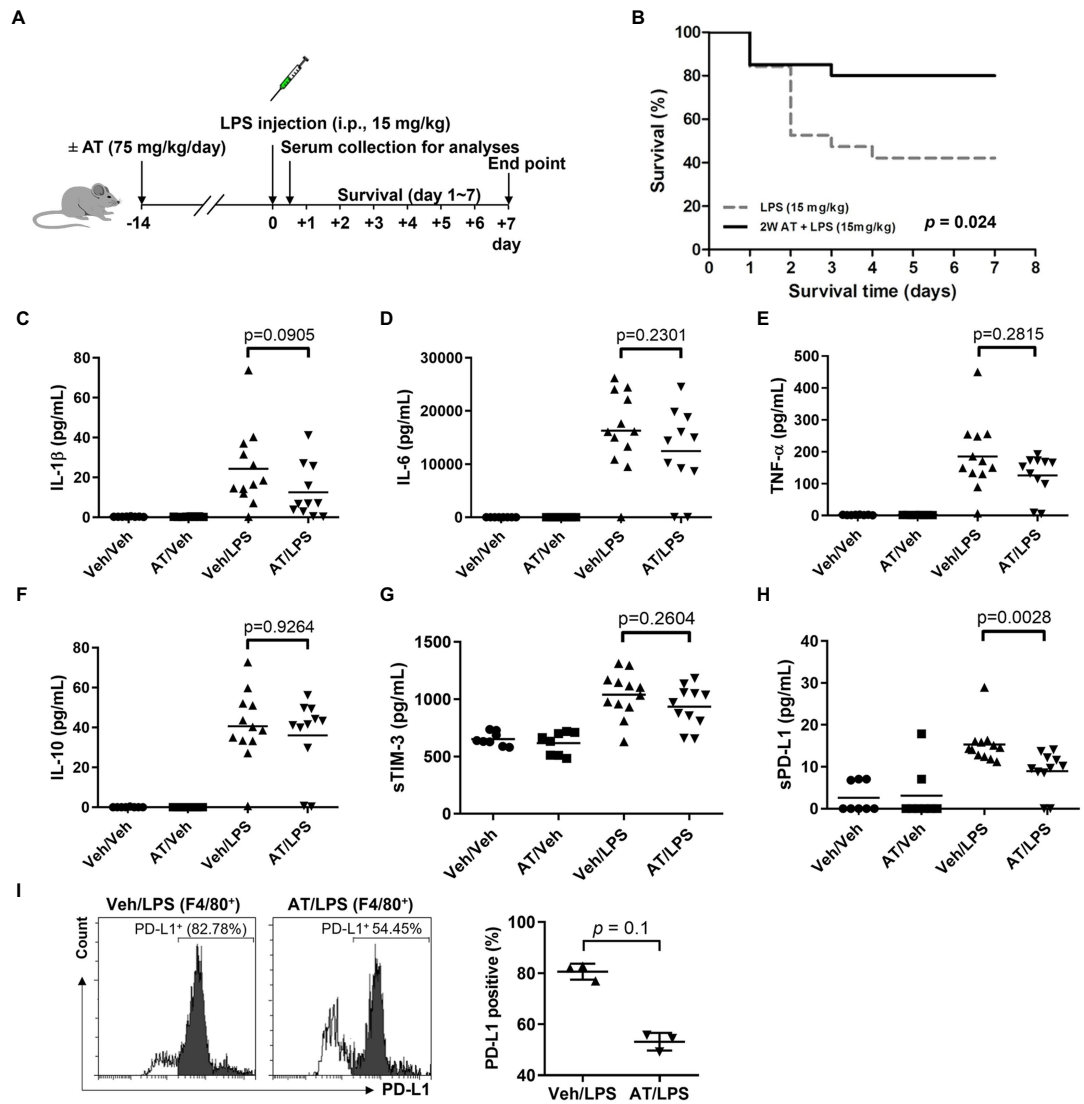
Effects of atenolol treatment on LPS-induced septic mice. **(A)** Schematic representation of experimental design. Two groups of C57BL/6 mice were injected with LPS (15 mg/kg) after 2 weeks of feeding with or without atenolol (75 mg/kg/day). **(B)** The Kaplan–Meier survival rate of LPS-induced septic mice with or without atenolol treatment. The mice were observed until day 7. **(C–I)** After feeding with or without atenolol for 2 weeks, C57BL/6 mice were injected with or without LPS (15 mg/kg). Samples were collected 12 h after vehicle or LPS injection. **(C–H)** Effects of atenolol treatment on the release of pro-inflammatory cytokines and soluble biomarkers in serum of LPS-induced septic mice. IL-1β, IL-6, TNF-α, IL-10, sTIM-3 and sPD-L1 were measured and analyzed by the ProcartaPlex multiplex assay. (I) The percentage of F4/80 positive macrophages expressing surface PD-L1 in BALF of LPS-induced septic mice. AT: atenolol, Veh: vehicle.

**Figure 2:**
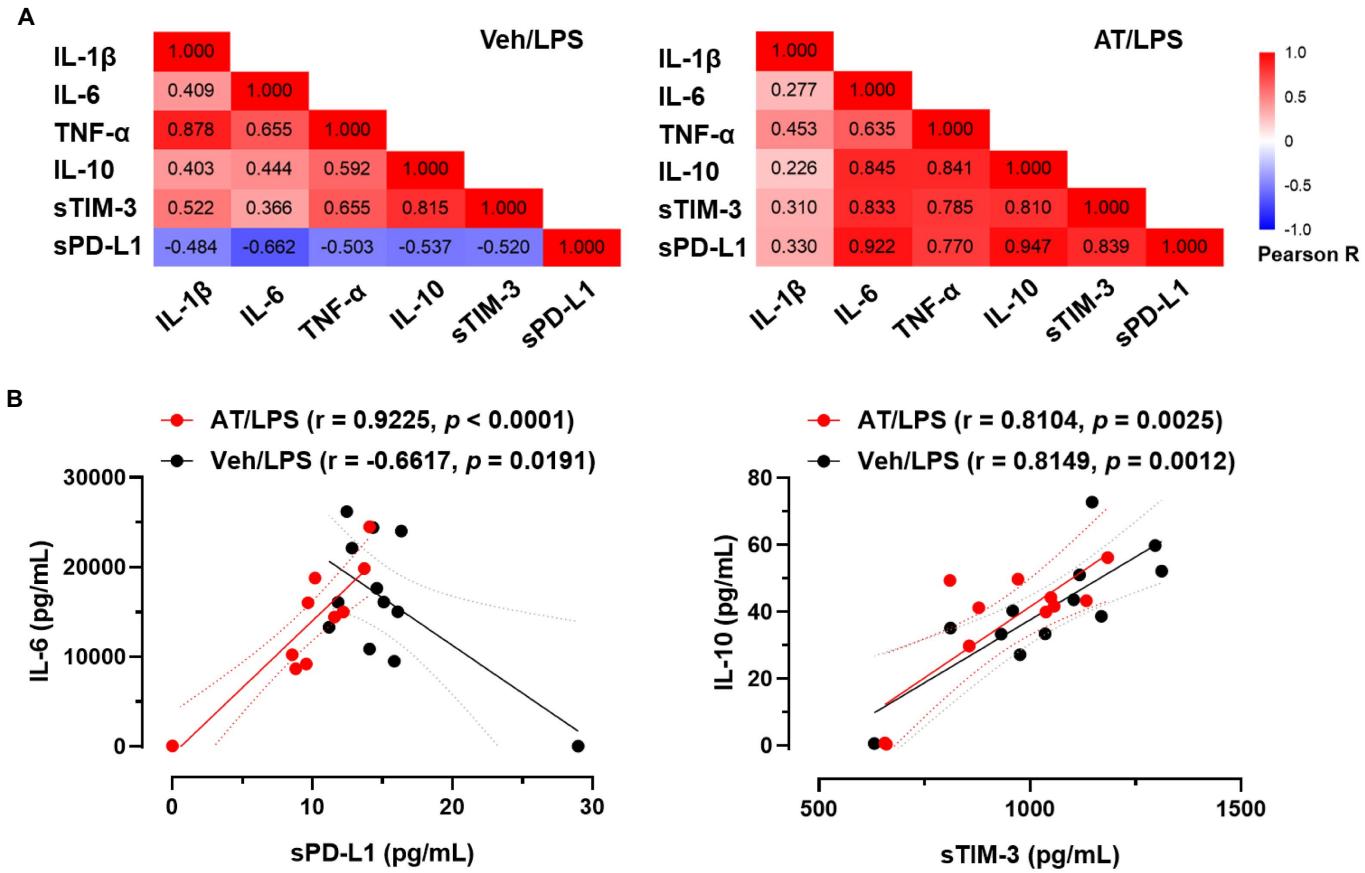
Effects of atenolol on the correlation between inflammatory cytokines and soluble immunomodulatory checkpoint molecules in LPS-induced septic mice. **(A)** Pearson correlation between inflammatory cytokines and sTIM-3 and sPD-L1 in septic mice. Differences in the correlation of individual cytokines and sPD-L1 were observed between septic mice treated with and without atenolol. **(B)** Correlation analysis of IL-6 and sPD-L1 or IL-10 and sTIM-3 in septic mice in the presence or absence of atenolol treatment.

As atenolol was recorded as a commonly used beta-blocker in the NHIRD database and can reduce the incidence of sepsis in patients, the effects of atenolol on septic mortality were further examined in an animal model (Figure 1A). We monitored mouse survival after LPS injection with or without atenolol pretreatment for 14 days. Based on Kaplan–Meier survival curves, mice with LPS-induced sepsis had a 7-day survival rate of 42%. The reduction in sudden death on the second day after LPS injection was particularly noticeable. Atenolol administration increased the 7-day survival rate to 80% relative to that in mice with un-pretreated sepsis (Figure 1B). The levels of cytokines, pro-inflammatory IL-1β, IL-6, TNF-α, and anti-inflammatory IL-10, were also strongly increased after 12 h from LPS stimulation relative to those measured in saline-injected control mice (Figures 1C–F). Even though statistical significance was not reached, atenolol administration partially attenuated the LPS-induced increase in IL-1β, IL-6 and TNF-α, while there was no effect on IL-10 release. Several studies have indicated that serum soluble programmed cell death 1 ligand (sPD-L1) and soluble T cell Ig and mucin domain protein 3 (sTIM-3) levels were positively correlated with the severity of sepsis and can be used as a biomarker to predict the immunosuppressive-induced mortality in sepsis (35–37). Interestingly, atenolol administration significantly reduced the sPD-L1 level and partially diminished the sTIM-3 level in LPS-induced septic mice (Figures 1G,H). Furthermore, in the septic mice that were pretreated with atenolol, there was a reduction in the percentage of F4/80 positive macrophages that expressed surface PD-L1, with a decrease of approximately 20%, although this change did not reach statistical significance (Figure 1I). Since sPD-L1 and sTIM-3 are involved in the regulation of immune tolerance, we compared the correlation of sPD-L1 and sTIM-3 levels with inflammatory cytokines. Regardless of atenolol treatment, sTIM-3 was positively correlated with inflammatory cytokines in septic mice and was statistically significant with IL-10 (Figure 2). Unexpectedly, sPD-L1 was negatively correlated with inflammatory cytokines and showed statistical significance with IL-6 in septic mice, whereas atenolol treatment reversed this phenomenon to a positive correlation (Figure 2). Atenolol thus alleviated the imbalance between pro-inflammation and anti-inflammation of the immune system. This suggests that atenolol-fed mice may have lower susceptibility to LPS-driven sepsis and reduced mortality during disease.

### 3.3. Atenolol downregulated ROS production and PD-L1 expression in monocytes/macrophages through the NF-κB and STAT3 pathways

**Figure 3:**
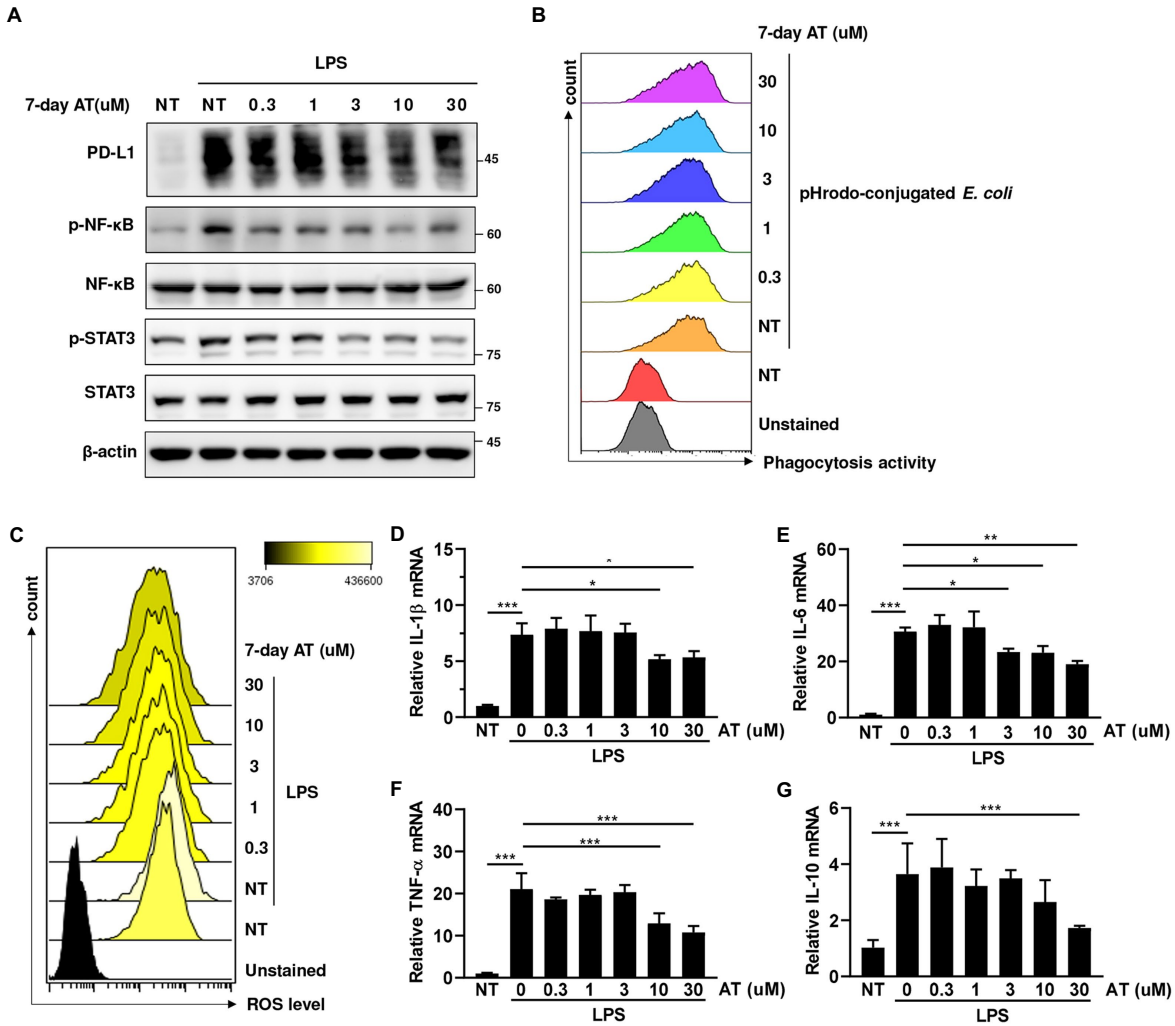
Effects of atenolol on the functions of LPS-induced monocyte/macrophages in THP-1 cells. **(A)** THP-1 cells were pretreated with indicated doses of atenolol for 7 days, and then stimulated with 100 ng/mL LPS overnight. The effect of atenolol on the level of PD-L1 and the activation of NF-κB and STAT3 were detected by immunoblotting. **(B)** Atenolol pretreated THP-1 cells were cocultured with pHrodo-conjugated *E. coli*, and then the phagocytosis activity was analyzed by flow cytometry. Atenolol pretreated THP-1 cells were cocultured with pHrodo-conjugated *E. coli*, and then the phagocytosis activity was analyzed by flow cytometry. **(C–G)** Following the same treatment as **(A)**, ROS production was detected by flow cytometry **(C)** and the expression of inflammatory-related cytokine IL-1β, IL-6, TNF-α, and IL-10 was detected by RT-qPCR **(D–G)**. *, *p*<0.05; **, *p*<0.01; ***, *p*<0.001.

Since the reduction of sudden death in the atenolol-treated group was particularly pronounced on the second day after LPS injection, we speculated that atenolol may have a regulatory role in innate immunity. Monocytes and monocyte-derived macrophages are key effectors and regulators of inflammation and innate immune responses. Considering that PD-L1 on monocytes/macrophages was upregulated in septic animals and PD-L1 blockade enhanced bacterial clearance and significantly improved their survival (38), we examined the effect of atenolol on PD-L1 expression in LPS-stimulated THP-1 cells. To simulate long-term use of drugs, we pretreated THP-1 cells with the indicated doses of atenolol for 7 days before LPS stimulation. Interestingly, atenolol treatment downregulated LPS-induced PD-L1 expression in monocytes/macrophages through the NF-κB and STAT3 pathways (Figure 3A). To understand whether atenolol affected LPS-induced dysfunction of monocyte/macrophages, we examined the pathogen clearance by THP-1 cells. However, atenolol had no effect on the phagocytic activity of monocytes/macrophages (Figure 3B). In contrast, atenolol treatment reduced the reactive oxygen species (ROS) level of THP-1 cells in a dose-dependent manner (Figure 3C). Although ROS play essential roles in monocyte/macrophage killing of pathogens (39), ROS production has also been recognized as one of the earliest innate immune responses of host cells in response to infection (40). Indeed, the expression of NF-κB and STAT3-dependent pro-inflammatory and anti-inflammatory genes were also attenuated by atenolol treatment (Figures 3D–G). These results suggest that atenolol was able to attenuate ROS-induced NF-κB and STAT3 activation, leading to a modest inflammatory response. Considering sPD-L1 may reflect PD-L1 levels expressed on the cell surface, our *in vitro* results also suggested that atenolol may regulate the expression of PD-L1 on monocytes/macrophages, which are the first-line innate immune cells to defend against infection.

## 4. Discussion

This nationwide population-based study was designed to investigate the impact of individual beta-blockers on the development of sepsis and to investigate whether the use of selective beta-blockers is associated with a reduced risk of sepsis. A reduction in risk of sepsis was consistently observed among the most commonly used selective beta-blockers, including atenolol, bisoprolol and metoprolol. We further found that atenolol downregulates ROS production and PD-L1 expression in monocytes/macrophages *via* the NF-κB and STAT3 pathways, which might maintain the immune homeostasis to improve survival in septic mice. To the best of our knowledge, this is the first study showing the beneficial effects of selective beta-blockers, particularly atenolol, on sepsis.

Over the past decades, several studies have investigated the effect of beta-blockers on sepsis among specific populations, but no consistent result has been found (25–28). Maier et al. reported that beta-blockers did not lower the risk of infection among patients after acute stroke (25, 26). In contrast, Sykora et al. showed that both pre-stroke and on-stroke beta-blocker therapy was associated with reduced frequency of pneumonia (adjusted risk ratio, 0.77; 95% CI, 0.6–0.98 and risk ratio, 0.49; 95% CI, 0.25–0.95) (27). Merli et al. reported that the use of beta-blockers had a protective effect against sepsis in patients with liver cirrhosis (OR, 0.46, 95% CI, 0.3–0.7) (28). However, none of these studies investigated the different effects between selective and non-selective beta-blockers. In this study, we found that the protective effect can be only seen in selective beta-blockers, but not in non-selective beta-blockers. This finding may help explain the controversial results reported in previous studies (25–28). However, given the observational nature of this study, further randomized trials would be necessary to confirm our findings.

Patients with sepsis often have fever, shock and respiratory failure due to an uncontrolled host immune response. A number of medications are used for treating sepsis and septic shock. The initial studies in sepsis immunotherapy were limited to blocking the hyperinflammatory phase of sepsis. More than 30 clinical trials have shown no beneficial effects of immunosuppressive therapies on sepsis, including anti-endotoxin molecules, TLR-receptor antagonists, anti-cytokine therapies (e.g., anti-TNF-α, IL-1Rα), and high dose corticosteroids (10). Nevertheless, some studies suggested that subgroups of sepsis patients with excessive inflammation may benefit from immunosuppressive therapy, such as IL-1 receptor blockade or anti-TNF neutralizing antibody (41). However, in some sepsis patients, severe conditions are induced, such as, immunoparalysis, which involves apoptosis of large numbers of T cells and B cells, hyposecretion of inflammatory cytokines following cytokine cascades and the dysfunction of immune cells. To date, most sepsis patients survive the initial proinflammatory phase but eventually die later during the immunoparalysis phase. In a systemic review of human and animal studies by Sanfilippo and Chacko (19, 24), beta-blockers were shown to modulate sepsis-induced alterations at the cardiovascular, metabolic, immunologic and coagulation levels. Atenolol is a synthetic selective blocker for beta-1-adrenergic receptors (ADRB1), and primarily used to treat hypertension and heart-associated chest pain (42). In our *in vitro* cell culture system, atenolol reduced ROS overproduction and its downstream NF-κB activation, which links the pro-inflammatory responses of alveolar macrophage in sepsis (43). Meanwhile, atenolol also attenuated STAT3 signaling which is known to exacerbate LPS-induced sepsis (44). Since both NF-κB and STAT3 have been found to regulate PD-L1 transcriptional activation (45), atenolol likely inhibits PD-L1 expression by inhibiting the activation of these two transcription factors. It has been reported that the expression of PD-L1 on monocytes/macrophages was increased in patients with sepsis (46, 47). These findings may also explain atenolol treatment reducing sPD-L1 in our *in vivo* septic mouse models, possibly due in part to lower expression of PD-L1 on monocytes/macrophages. However, more evidence will be needed to verify our findings.

This study has several strengths. It is a relatively large cohort study conducted in a real-world setting. The use of a nationwide population-based database, in contrast to a hospital-based database, minimizes the risk of selection bias and increased generalizability. In addition, we conducted a series of *in vivo* and *in vitro* experiments to validate our real-world findings and explore the underlying mechanism.

This study also has several limitations. First, we did not have detailed data such as basic data like body length, body weight, blood pressure, and laboratory tests. Therefore, we could not evaluate the effect of beta-blocker usage on immune cells, the severity of hypertension and whether every patient received appropriate anti-hypertensive treatment according to the recommended guidelines. Second, this study was not a randomized controlled study. Third, the mice LPS model is not a good model to represent human sepsis, especially in terms of survival, as this model represents more of a toxic shock rather than sepsis. Another limitation is that mice used in this model are relatively young, whereas patients who are chronically prescribed beta blockers are often older and have comorbidities. Nonetheless, our findings derived from real-world data and validated in the laboratory are more likely to be reflective of common clinical practice.

## 5. Conclusion

Premorbid treatment of selective beta-blockers, such as atenolol, was associated with a reduced incidence of sepsis in hypertension patients. Furthermore, atenolol administration improved survival in septic mice by modulating immune homeostasis and downregulated ROS production and PD-L1 expression in monocytes/macrophages *via* the NF-κB and STAT3 pathways.

## Data availability statement

The datasets used and analyzed in the current study are available from the corresponding author on reasonable request.

## Ethics statement

This study involving human data was approved by the Institutional Review Board of the National Health Research Institutes (Taiwan) with a waiver of informed consent (protocol no. EC1011008-E). All procedures were followed in accordance with the ethical standards of the Institutional Review Board of the National Health Research Institutes and with the Helsinki Declaration of 1975. All animal operations were executed in compliance with the applicable regulations and approved by the Institutional Animal Care and Use Committee of Cardinal Tien Hospital, New Taipei, Taiwan (protocol no. 107A-006).

## Author contributions

C-CL and C-YW: conceptualization. S-YH, N-CT, N-JC, and YL: data curation. S-YH, N-CT, N-JC, and Y-HW: formal analysis. S-YH, C-CL, C-HC, C-CH, and YL: investigation. S-YH, C-HC, C-CH, Y-HW, and YL: methodology. S-YH, Y-HW, YL, C-YW, and LC: funding acquisition. S-YH, C-CL, Y-HW, YL, and C-YW: writing and editing. C-CL, C-YW, and LC: project administration and supervision. All authors contributed to the article and approved the submitted version.

## Funding

This work was supported by grants from the Ministry of Science and Technology of Taiwan (MOST 105-2320-B-038-055-MY2, MOST 110-2314-B-567-004, and MOST 111-2314-B-567-003), Cardinal Tien Hospital (CTH 110A-2201, CTH 110A-2206 and CTH 111A-2201), and the National Health Research Institutes (intramural funding).

## Conflict of interest

The authors declare that the research was conducted in the absence of any commercial or financial relationships that could be construed as a potential conflict of interest.

## Publisher’s note

All claims expressed in this article are solely those of the authors and do not necessarily represent those of their affiliated organizations, or those of the publisher, the editors and the reviewers. Any product that may be evaluated in this article, or claim that may be made by its manufacturer, is not guaranteed or endorsed by the publisher.
